# VEGF-VEGFR Signaling Mechanism Directs the Migration of Newborn Hemocytes from the Hematopoietic Site of Oyster *Crassostrea gigas*

**DOI:** 10.3390/cells14181446

**Published:** 2025-09-16

**Authors:** Simiao Yu, Miren Dong, Xue Qiao, Yuhao Jin, Xiyang Liu, Muchun He, Lingling Wang, Linsheng Song

**Affiliations:** 1 Liaoning Key Laboratory of Marine Animal Immunology and Disease Control, Dalian Ocean University, Dalian 116023, China; yusimiao1029@163.com (S.Y.); dongmiren723@163.com (M.D.); qiaoxue@dlou.edu.cn (X.Q.); 18853858802@163.com (Y.J.); lxy18228381382@163.com (X.L.); hemuchun@dlou.edu.cn (M.H.); 2 Laboratory of Marine Fisheries Science and Food Production Process, Qingdao Marine Science and Technology Center, Qingdao 266237, China; 3 Dalian Key Laboratory of Aquatic Animal Disease Prevention and Control, Dalian Ocean University, Dalian 116023, China; 4 Southern Marine Science and Engineering Guangdong Laboratory (Zhuhai), Zhuhai 519000, China

**Keywords:** hematopoietic site, vascular endothelial growth factor, cell migration, cell proliferation, *Crassostrea gigas*

## Abstract

Hematopoiesis is a complex process of creating new hemocytes and releasing them from hematopoietic tissue. In the present study, the hematopoietic site in oyster *Crassostrea gigas* was successfully identified in the proximal sector (designated G2–G3) of the gill hinge with a substantial number of newborn cells and a minor presence of stem-like cells. The homologues of VEGF (*Cg*VEGF) and its receptor *Cg*VEGFR were characterized, and they interacted with each other. After the oysters received an injection of r*Cg*VEGF, the number of EdU-positive (EdU^+^) cells increased within the G2–G3 sector and the hemolymph. When the expression of *Cg*VEGFR was inhibited by RNAi, the percentage of EdU^+^ cells in the hemolymph declined dramatically, but increased significantly in the G2–G3 sector and EdU^+^ cells aggregated in this region. Meanwhile, the phosphorylation levels of *Cg*Erk and *Cg*JNK, mRNA transcripts of cell proliferation-related and cell migration-related genes, reduced significantly. These results indicate that the proximal region of the hinge in gill was the site producing hemocytes, and *Cg*VEGF-VEGFR-MAPK signaling pathway induced the migration of newborn hemocytes from this site to the circulating hemolymph, which provides new clues about hematopoiesis in primary invertebrates.

## 1. Introduction

Hematopoiesis is a continuous orchestrated process of proliferation, self-renewal, and differentiation of hematopoietic stem cells (HSCs) in hematopoietic tissues, which contributes significantly to the establishment and maintenance of the blood system, as well as the immune system [1]. Cells that originate in the hematopoietic tissue migrate to the target tissues to differentiate into mature immune cells and then they are exported to the periphery to execute immune functions. The hematopoietic tissues provide an efficient microenvironment with multiple extracellular and intracellular components for the strict regulation of proliferation, differentiation, and migration of these cells [2]. Hematopoietic tissue has a long evolutionary history and its location varies among different species. In mammals, the sequential sites of hematopoiesis change during development, including the yolk sac, an area surrounding the dorsal aorta termed the aorta–gonad mesonephros (AGM) region, the fetal liver, and finally the thymus and bone marrow [3]. The thymus appears in all jawed vertebrates, but its location differs among species from different evolutionary taxa. In the most primitive jawed vertebrate cartilaginous fish and the jawless vertebrate lamprey, the thymus or thymic precursor tissue seems to be distributed in the gill filaments [4]. Meanwhile, in invertebrates, due to the extremely vast species diversity and the primitive nature of the immune system, the hematopoietic tissues/sites vary greatly. The axial organ and the pharynx have been considered as hematopoietic sites in the sea urchin (*Strongylocentrotus purpuratus*) [5]. In Drosophila, hematopoiesis occurs in the lymph gland of larvae and in the dorsal abdominal hemocyte clusters of adults [6]. In crayfish *Pacifastacus leniusculus*, the newborn hemocytes were observed in a separate sheet of cell clusters (lobes) situated on the dorsal side of the stomach [7]. In mollusks, hemocytes are thought to originate from the white body in cephalopods, the pericardial region in gastropods, as well as the gill in more primitive bivalves [8]. Investigating the production and migration of newborn cells in bivalves is necessary to provide essential clues for understanding the hematopoiesis mechanism and tracing the origin of hematopoietic tissue in invertebrates.

Recent advances have improved our understanding of the HSC niche, which is a locally specialized microenvironment that maintains and regulates HSCs’ proliferation, differentiation, and especially their migration into the circulating system [9]. The molecular components of the HSC niche, such as pro-hematopoietic growth factors, chemokines, and cytokines have been identified in the bone marrow of mammals [2]. Vascular endothelial growth factor (VEGF) is a potent and multifunctional cytokine that regulates multiple cellular responses after binding to its receptor (vascular endothelial growth factor receptor, VEGFR). In mice, VEGF and VEGFR have been reported to regulate the differentiation and mobilization of HSCs [10]. It is generally believed that the VEGF pathway is a relatively conserved regulatory mechanism associated with cell proliferation and migration in invertebrates. PVF/PVR (the homologues of VEGF and VEGFR) have been reported to be involved in the proliferation, differentiation, and migration of intestinal epithelium, and hyperproliferation of hemocytes in Drosophila [11]. The PVF/PVR pathway participates in the control of hematopoietic progenitor cell migration in crayfish *P. leniusculus* [12]. The regulation mechanism of those cytokines in the HSC niche in invertebrates remains incompletely defined and beset by limited models.

The Pacific oyster *Crassostrea gigas* is a sessile marine invertebrate inhabiting the estuarine and intertidal regions abundant in microbial challenges [13], and thus provides an ideal model for studying the evolution of the immune system. The hemocytes produced from hematopoiesis are the core component of the immune system, and are crucial for protecting oysters against microbial infection. The irregularly folded structure in the gill has been suggested as the possible hematopoietic site of adult oysters [14]. However, the production and migration mechanisms of newborn cells in the hematopoietic site of oysters are largely unknown. In the present study, the homologues of VEGF (*Cg*VEGF) and VEGFR (*Cg*VEGFR) were identified from *C. gigas* with the following main objectives: (1) to identify and characterize the different sectors of the gill for the localization of potential hematopoietic site and monitor the expression patterns of *Cg*VEGF and *Cg*VEGFR during the immune response, (2) to explore the regulation of *Cg*VEGF on cell proliferation, and (3) to clarify the possible role of *Cg*VEGF and *Cg*VEGFR-mediated signal in the cell migration of oysters.

## 2. Materials and Methods

### 2.1. Animals and Microbes

Adult Pacific oysters of the *C. gigas* variety, averaging 13.0 ± 0.5 cm in shell length and 180 ± 10 g in weight, were sourced from a commercial aquaculture facility located in Dalian, Liaoning Province, China. The bacterial strain *Vibrio splendidus* (preserved in our laboratory) was cultured in 2216E marine broth at 16 °C for 24 h. The bacterial suspension was then adjusted to a final concentration of 2 × 10^8^ CFU/mL for immune stimulation. All animal experiments were performed according to protocols approved by the Ethics Committee of Dalian Ocean University.

### 2.2. Immune Stimulation and Sample Collection

**Figure 1:**
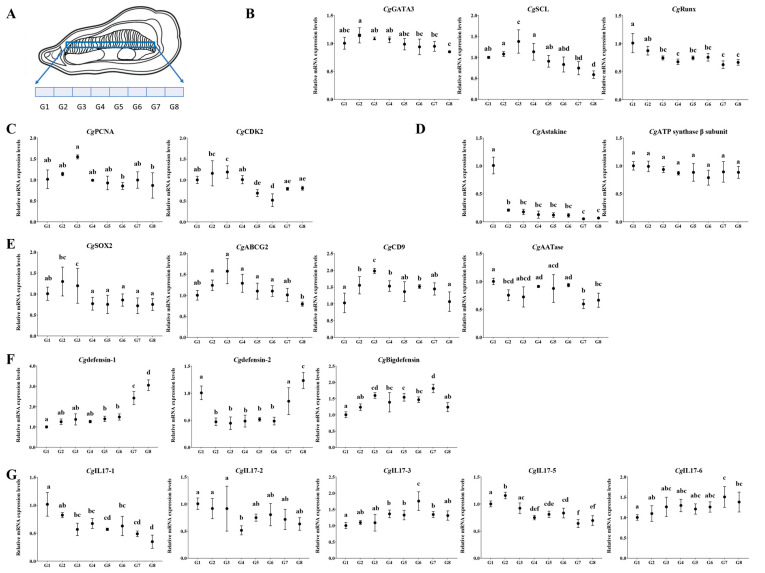
The transcription pattern of hematopoiesis- and immune-related molecules in different sectors of the gill. (**A**) Schematic representation of eight sectors of the gill. The sectors were named G1 to G8 from the proximal region of the hinge to the distal region. (**B**–**G**) The mRNA expression level of hematopoietic transcription factors and immune-related molecules in different regions of gills quantified via RT-qPCR, normalized to *Cg*EF. Vertical bars are shown as the mean ± S.D. (N = 3). The different letters show the significant differences compared with other groups (*p* < 0.05) as determined by one-way ANOVA with Dunnett’s post hoc test.

The oysters were cultured in aerated seawater at 15 ± 2 °C for 14 days before processing. The gill tissues were individually collected from nine oysters (randomly assigned to three biological pools) and uniformly classified into eight sectors according to anatomical location. The sectors were named G1 to G8 based on their position, from close by to further away from the hinge (Figure 1). Hemolymph and various tissues were harvested from nine additional oysters via the same protocol [15]. A total of 150 oysters were subjected to immune stimulation according to a previously established protocol [16]. Briefly, they were randomly divided into *V. splendidus* (VS) and seawater (SW) groups and injected with 100 μL of bacterial suspension and an equivalent volume of sterile seawater, respectively. Gill tissues (G2–G3 sector) were sampled from nine oysters with three replicates for each time point, including 0, 3, 6, 12, 24, 48, and 72 h after *V. splendidus* stimulation. All the collected samples were immediately homogenized in 1 mL of TRIzol^TM^ reagent for total RNA extraction.

To assess cell proliferation dynamics in response to immune challenge and recombinant *Cg*VEGF protein (r*Cg*VEGF) treatment, a total of 36 oysters were randomly assigned to two experimental cohorts. The first cohort was designated for the *V. splendidus* challenge, with an SW group serving as the control group. The second cohort received r*Cg*VEGF treatment, with a recombinant GST (rGST) group as the corresponding control group. All oysters received an initial intramuscular injection of 100 μL of 0.2 mM EdU. Twelve hours later, the first cohort received a second injection of either 100 μL of *V. splendidus* suspension (2 × 10^8^ CFU/mL) or an equivalent volume of sterile seawater. Concurrently, the second cohort received a second injection of either 100 μL of r*Cg*VEGF (0.2 mg/mL) or 100 μL of rGST protein (0.2 mg/mL). Following the second injection, hemocytes and gill tissues (G2–G3 sector) were collected from nine oysters with three replicates. Gill samples were immediately fixed in modified Bouin’s fluid for subsequent histological analysis. The percentage of EdU-positive cells (EdU^+^ cells) in hemocytes was quantified using flow cytometry, strictly adhering to the manufacturer’s protocol.

### 2.3. Bio-Layer Interferometry Assay

The recombinant proteins of r*Cg*VEGF and r*Cg*VEGFR were produced and purified following an established protocol, with specific modifications [17]. Codon-optimized genes encoding *Cg*VEGF and *Cg*VEGFR (Sangon Biotech, Shanghai, China) were cloned into pGEX-4T-1 and pET-30a expression vectors, respectively, and transformed into *Escherichia coli* Transetta (DE3) for protein production. Recombinant protein expression was induced with 0.25 mM IPTG at an OD_600_ of 0.6 for 6 h. The r*Cg*VEGF and r*Cg*VEGFR proteins were purified by GST and His-tag affinity chromatography as per the manufacturer’s instructions.

The binding kinetics between r*Cg*VEGF and r*Cg*VEGFR were analyzed by Bio-Layer Interferometry (BLI) on an Octet K2 system (ForteBio). Biotinylated r*Cg*VEGF was immobilized onto streptavidin biosensors and exposed to a two-fold serial dilution series of r*Cg*VEGFR (1150 μg/mL, 1125 μg/mL, 843.75 μg/mL, 632.81 μg/mL, and 474.61 μg/mL). The equilibrium dissociation constant (KD) was derived by globally fitting the association and dissociation curves to a 1:1 binding model using ForteBio software v12.0 (ForteBio, Fremont, CA, USA).

### 2.4. RNA Interference (RNAi) of CgVEGFR with siRNA

To investigate the role of *Cg*VEGFR in VEGF-mediated cell proliferation, a targeted knockdown was performed using specific siRNAs (synthesized by Shanghai Jima Biotechnology Co., Ltd., Shanghai, China) designed as previously described [18]. Briefly, oysters were randomly assigned to three experimental groups, si-*Cg*VEGFR + r*Cg*VEGF, si-NC + r*Cg*VEGF, and a SW control group. The injection protocol was as follows. All oysters received an initial co-injection of 100 μL of the respective siRNA (1 OD in seawater) or SW, along with 100 μL of 0.2 mM EdU. A second injection of the same siRNA or SW was administered 12 h later. At 12 h post the second injection, a third injection of 100 μL r*Cg*VEGF (0.2 mg/mL in seawater) was given to the si-*Cg*VEGFR + r*Cg*VEGF and si-NC + r*Cg*VEGF groups, while the SW group received an equivalent volume of seawater. Hemocytes and gill tissues (G2–G3 sector) were collected from each group 6 h after the final injection. The EdU-positive signals labeled with Alexa Fluor 488 (green fluorescence signal) or 647 (red fluorescence signal) (Beyotime, Shanghai, China) in hemocytes were detected and calculated by flow cytometry according to the protocol of the manufacturer. The gill samples were divided equally into three parts for immunohistochemistry, RNA and protein analyses.

VEGF activator Sodium taurocholate (STC, MedChemExpress, Monmouth Junction, NJ, USA) was used for the activation experiment in si-*Cg*VEGFR oysters. In the si-*Cg*VEGFR + STC group and si-NC + STC group, oysters were separately subjected to interference by two successive injections with siRNA or si-NC, and then an injection of 100 μL of STC (0.2 mg/mL in seawater) at 12 h after the second injection as described above. The treatment for the SW group and the sample collection for each group were conducted as described above.

### 2.5. The Inhibition of CgVEGFR Expression by the Treatment with Inhibitors

The percentage of EdU+ cells in the total circulating hemocytes, the protein abundance of *Cg*VEGFR and phosphorylated MAPK pathway genes, the mRNA expression levels of cell proliferation-related and cell migration-related genes, and the distribution of EdU+ cells in G2–G3 sector were investigated after the expression of *Cg*VEGFR was inhibited by Semaxanib (VEGFR inhibitor, Beyotime, Shanghai, China) and Brivanib (VEGFR inhibitor, Beyotime, Shanghai, China). Semaxanib and Brivanib were first dissolved in DMSO (MedChemExpress, Monmouth Junction, NJ, USA), and then diluted with seawater at a final concentration of 0.5 mg/mL. In the Semaxanib + r*Cg*VEGF group and Brivanib + r*Cg*VEGF group, the oysters first received an injection of 100 μL diluted inhibitors and 100 μL 0.2 mM of diluted EdU, and then another injection of 100 μL r*Cg*VEGF (0.2 mg/mL in seawater) at 12 h after the injection with inhibitors. In the DMSO + r*Cg*VEGF group, the oysters first received an injection of 100 μL of diluted DMSO and 100 μL of 0.2 mM diluted EdU, and then another injection of 100 μL r*Cg*VEGF (0.2 mg/mL in seawater) at 12 h after EdU injection. At 6 h after the final injection, the hemocytes and G2–G3 sector of the gill were collected from each group for assays as previously mentioned.

### 2.6. Reverse Transcription Quantitative PCR (RT-qPCR) Analysis

Total RNA was extracted using Trizol^TM^ reagent (Thermo Fisher Scientific, Waltham, MA, USA) in strict accordance with the prescribed protocol. Subsequently, RT-qPCR was employed to quantify the relative mRNA expression levels of the target genes, with the *Cg*EF gene (NP_001292242.2) serving as the endogenous control for normalization. The PCR reactions were performed on an ABI QuantStudio sequence detection system (Applied Biosystems, Waltham, MA, USA) utilizing SYBR Premix Ex Taq (Takara, Kyoto, Japan). The specificity of the amplification was verified by dissociation curve analysis at the conclusion of each run, ensuring the presence of a single, specific product. Relative mRNA expression levels were calculated using the 2^−∆∆CT^ method and are presented as the mean ± standard deviation (S.D.) of three independent biological replicates (N = 3) [19]. The primer sequences used in this study are provided in Supplementary Table S1.

### 2.7. Histochemical Staining and Immunohistochemistry Assay of Gill Sections

Histochemical and immunohistochemical assay was conducted as previously reported [20]. In brief, gill tissues were fixed in modified Bouin’s fluid at room temperature for 24 h, followed by dehydration through a graded ethanol series. The dehydrated samples were then embedded in paraffin in a transverse orientation and serially sectioned at 4 µm thickness. After baking at 52 °C overnight, the sections were rehydrated through a descending ethanol series. Histochemical staining was performed using a commercial kit (Solarbio, Beijing, China) according to the manufacturer’s instructions. Finally, the sections were mounted with neutral balsam and observed under a light microscope. For digital image analysis, the stained slides were scanned using a Pannoramic Digital Slide Scanner (3DHISTECH, Budapest, Hungary), and the resulting images were analyzed with CaseViewer software v2.4 (3DHISTECH, Budapest, Hungary).

To identify newly generated hemocytes within hematopoietic tissues, gill sections were subjected to EdU staining (Thermo Fisher Scientific, Waltham, MA, USA) per the manufacturer’s instructions. Briefly, sections were fixed in 4% paraformaldehyde for 15 min, permeabilized with 0.5% Triton X-100 in PBS for 15 min, and incubated with the EdU reaction cocktail (500 μg/mL CuSO_4_, 40 μM Alexa Fluor 488 Azide, and Click Reaction and Additive Solution) for 30 min. After washing three times with PBST, the sections were stained with DAPI (Beyotime, Shanghai, China) for 5 min to counterstain cell nuclei, and observed under a fluorescence microscope (Axio Imager A2, Zeiss, Oberkochen, Germany).

Immunohistochemical localization of SOX2 and detection of cell proliferation via EdU labeling were performed sequentially on the gill sections. EdU staining was initially executed using the BeyoClick™ EdU Kit (Beyotime, Shanghai, China) with Alexa Fluor 647. This was followed by incubation with a rabbit anti-SOX2 primary antibody (Abcam, Cambridge, UK) at 37 °C for 1 h and subsequently with an Alexa Fluor 488-conjugated goat anti-rabbit IgG secondary antibody (Thermo Fisher Scientific, Waltham, MA, USA) at 37 °C for 50 min, adhering to a published protocol [14]. Cell nuclei were counterstained with DAPI for 5 min, and fluorescent signals were captured using an Axio Imager A2 microscope (Zeiss, Oberkochen, Germany).

### 2.8. Western Blotting Analysis

Western blotting assay was carried out as described previously, with minor modifications [21]. The specificity of the antibodies against ErK, P38, JNK, p-ErK, p-P38, p-JNK (Abcam, Cambridge, UK) and GAPDH (Proteintech, Chicago, IL, USA) was confirmed in our prior work [17] and in this study before use. Gill lysates were prepared in RIPA buffer (Beyotime, Shanghai, China), and clarified supernatants were resolved by SDS-PAGE and electrotransferred onto membranes. The membranes were probed overnight at 4 °C with primary antibodies, including anti-ErK, anti-p38, anti-JNK, anti-p-ERK, anti-p-p38, and anti-p-JNK (all from Abcam, Cambridge, UK), and anti-GAPDH (Proteintech, Chicago, IL, USA), all diluted at 1:1000. After washing, the membranes were incubated with a 1:1000 dilution of HRP-conjugated secondary antibody (Beyotime, Shanghai, China). Protein bands were visualized using enhanced chemiluminescence (ECL) reagent (Beyotime, Shanghai, China) and imaged with Amersham Imager 600 (GE Healthcare, Chicago, IL, USA). The relative protein expression levels were quantified by analyzing the band intensity with ImageJ 6.7 software.

### 2.9. The Flow Cytometry Analysis

Hemocyte proliferation was quantified using the BeyoClick™ EdU Kit (Beyotime, Shanghai, China) on a FACS Aria II flow cytometer (BD Biosciences, Franklin Lakes, NJ, USA), according to the manufacturer’s protocol with minor modifications [22] as outlined above. A sequential gating strategy was employed for the analysis of EdU-positive cells. First, we used forward scatter-area (FSC-A) versus side scatter-area (SSC-A) to gate the hemocyte population, excluding debris and cell aggregates. Subsequently, single cells were selected from this population using a forward scatter-height (FSC-H) versus forward scatter-area (FSC-A) plot to exclude doublets or clumped cells. A negative control sample (untreated without the EdU detection reagent) was used to define the threshold for EdU-positive signals. The same gating strategy and threshold were then applied to all experimental samples to quantify the percentage of EdU-positive cells within the single, live hemocyte population for each group.

### 2.10. Statistical Analysis

All experimental data are presented as the mean ± standard deviation (SD). Statistical comparisons between two groups were conducted using a two-sample Student’s *t*-test. For comparisons among multiple groups, one-way analysis of variance (ANOVA) was employed, followed by Tukey’s post hoc test. All statistical analyses were performed using SPSS 26.0 software (IBM, Chicago, IL, USA). Statistical significance was defined as *p* < 0.05, and high significance as *p* < 0.01.

## 3. Results

### 3.1. The Proximal Region of the Gill Hinge Is the Hematopoietic Site in Oysters

The gill of oysters was divided into eight sectors, which were named G1–G8 (Figure 1A), respectively. The mRNA transcripts of hematopoiesis- and immune-related genes in different sectors of the gill were examined by RT-qPCR. The mRNA expression levels of hematopoietic transcription factors (*Cg*GATA3 and *Cg*Runx were higher in the G2–G3 sector, and *Cg*SCL was higher in the G1–G3 sector) were relatively higher in the G2 and G3 sectors compared to the other sectors (Figure 1B). The mRNA transcripts of cell cycle-related genes (*Cg*PCNA and *Cg*CDK2) showed relatively higher expression in G1 and G2 sectors (Figure 1C). The mRNA transcripts of hematopoiesis-related cytokine (*Cg*Astakine) showed relatively higher expression in the G1 sector, while there was no significant difference in the expression level of its receptor (*Cg*ATP synthase β subunit) (Figure 1D). The homologues of mammalian stem cell markers (*Cg*SOX2 and *Cg*ABCG2) were observed with relatively higher (*p* < 0.05) mRNA expression in sectors G2 and G3. The previously identified marker for oyster agranulocytes (*Cg*CD9) showed relatively higher (*p* < 0.05) expression in the G3 sector, while no significant difference was found in the expression of molecular markers for oyster granulocytes (*Cg*AATase) among the different sectors (Figure 1E). The mRNA expression levels of defensin superfamily members (*Cg*defensin-1, *Cg*defensin-2 and *Cg*Bigdefensin) were relatively higher (*p* < 0.05) in G7 and G8 sectors (Figure 1F), while that of Interleukin 17 (IL-17) subfamily members (*Cg*IL17-1, *Cg*IL17-2, *Cg*IL17-3, *Cg*IL17-5 and *Cg*IL17-6) were relatively higher (*p* < 0.05) in G1, G2 and G7 sector (Figure 1G).

### 3.2. The Generation of Newborn Cells Occurs at the Edge of Normal Gill Filaments in the Inner Demi-Branch of G2–G3 Sector

The structure of G2–G3 sector were observed after Histochemistry staining with Hematoxylin–Eosin and EdU fluorescent dyes. The nuclei were stained in purple, and there were a population of small, round cells with a large nucleus and thin cytoplasm observed in the vessels of the G2–G3 sector (Figure S1A). These small, round cells were intensely concentrated in the epithelium and the extremities of the tubules. The newborn hemocytes were labeled with EdU in red, and the nuclei were dyed by DAPI in blue. There were a large number of EdU-positive cells in the vessels of G2–G3 sector (Figure S1B).

**Figure 2:**
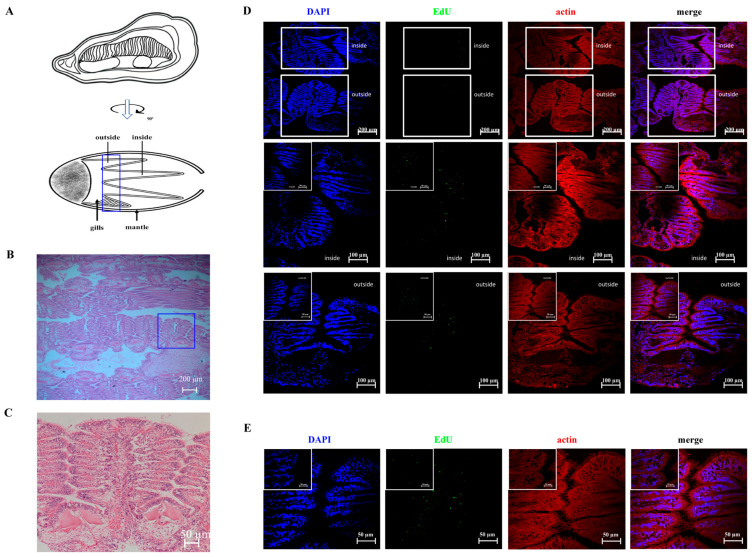
The structural characteristics of different demi-branches and filaments in the G2–G3 sector. (**A**) A schematic representation of different demi-branches in the G2–G3 sector. (**B**) The horizontal sector of a demi-branch stained by HE. Bar: 200 μm. (**C**) The horizontal sector of a filament cluster stained by HE. Bar: 50 μm. (**D**) The horizontal sector of a demi-branch stained by EdU. Bar: 200 μm or 100 μm. And Bar: 50 μm in enlarged image. (**E**) The horizontal sector of the filament cluster stained by EdU. The EdU-positive signals labeled with Alexa Fluor 488 are shown in green, and the cell nuclei are stained by DAPI (blue fluorescence). Bar: 50 μm. And Bar: 20 μm in enlarged image.

Four inner and outer demi-branches were named according to their locations (Figure 2A). The demi-branches in G2–G3 sector were further stained with HE, EdU, and DAPI. The demi-branch was found to be composed of simple filaments in clusters (Figure 2B), and there was one principal gill filament characterized by chitin-based scaffolds and several normal gill filaments in each cluster (Figure 2C). After HE staining, the nucleus and cytoplasm were observed in blue and pink, respectively. The newborn cells indicated by EdU were observed in green and the nuclei were stained by DAPI in blue. The positive green signals were partially distributed in the inner demi-branch (Figure 2D) and extensively distributed in the edges of gill filaments in normal oysters (Figure 2E).

### 3.3. The Green Signals of SOX2 Are Colocalized with Newborn Cells in the Hematopoietic Site

**Figure 3:**
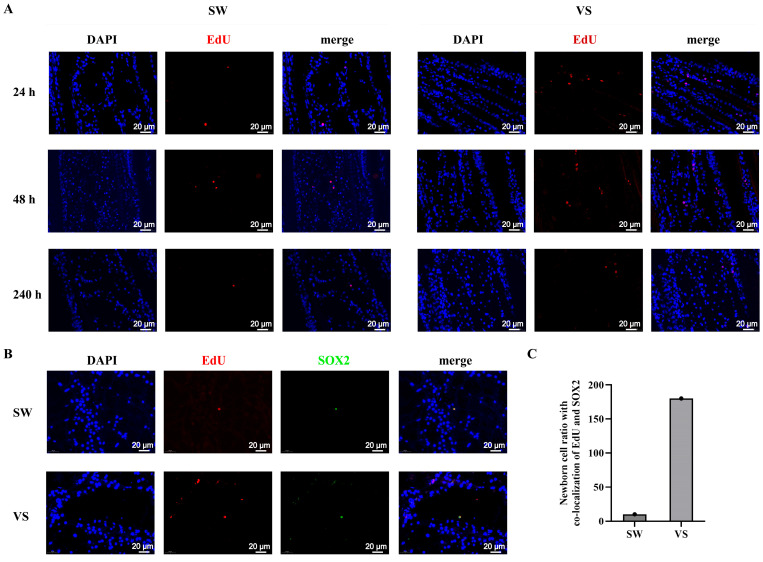
The EdU^+^ signals and colocalized *Cg*SOX2 in the G2–G3 sector after *V. splendidus* stimulation. (**A**) The EdU^+^ cells detected by immunofluorescence assay in the G2–G3 sector at 24, 48, and 240 h after *V. splendidus* stimulation. The EdU-stained cells are dyed by the red signals and the blue signals of DAPI indicates nuclei. (**B**) Immunofluorescence colocalized SOX2 and EdU^+^ cells in G2–G3 sector at 240 h after *V. splendidus* stimulation. The SOX2-positive signals labeled with Alexa Fluor 488 are shown in green, and the EdU-positive signals labeled with Alexa Fluor 647 are shown in red. The cell nuclei are stained by DAPI (blue fluorescence). Bar: 20 µm. (**C**) Newborn cells ratio with *Cg*SOX2 and EdU co-location analyzed by the Pearson correlation coefficient (×100). SW: Seawater; VS: *V. splendidus*.

An immunofluorescence assay was performed to detect the EdU-positive cells in G2–G3 sector after *V. splendidus* stimulation. The newborn cells labeled by EdU were observed in red signals with blue nuclei dyed by DAPI, which distributed in the tubule lumen region of gill vessels. At 24 and 48 h after EdU injection, the red fluorescence signal of EdU was observed to be bright and rich, and it became weaker but still observable at the tubule lumen region of gill vessels 240 h after EdU injection (Figure 3A). The fluorescence intensity of the red fluorescence increased in G2–G3 sector 24, 48, and 240 h after *V. splendidus* stimulation.

The distribution of cell stemness protein SOX2 in G2–G3 sector was observed by an immunofluorescence assay with the commercial rabbit-anti-SOX2 (Abcam, USA). The positive signal of *Cg*SOX2 was observed in green, distributed in the G2–G3 sector. At 240 h after EdU injection, the positive red fluorescence signals of EdU were observed in the tubule lumen region of gill vessels, and parts of them were colocalized with the green signals of *Cg*SOX2 (Figure 3B,C). After *V. splendidus* stimulation, the fluorescence intensity of the red fluorescence increased in G2–G3 sector until 240 h post EdU injection.

### 3.4. CgVEGF and CgVEGFR Are Highly Expressed in the G2–G3 Sector and Circulating Hemocytes, and Their Expression Levels Increase After V. splendidus Stimulation

The mRNA transcripts of *Cg*VEGF and *Cg*VEGFR were detected in all the examined tissues and sectors of gills. The expression level of *Cg*VEGF mRNA was higher in gills and hemocytes: 4.91-fold and 2.80-fold of that in gonads (*p* < 0.05), while the expression level of *Cg*VEGFR was higher in labial palp: 3.01-fold and 4.65-fold of that in gills and hemocytes (*p* < 0.05), respectively (Figure S2A). The mRNA transcripts of *Cg*VEGF were universally distributed in all the sectors without a significant difference (*p* > 0.05), while the expression level of *Cg*VEGFR was higher in the G2 sector than in other sectors (*p* < 0.05) (Figure S2B).

The mRNA expression levels of *Cg*VEGF and *Cg*VEGFR in G2–G3 sector were further examined after *V. splendidus* stimulation. The expression level of *Cg*VEGF was upregulated significantly 6, 12, 24, 48, and 72 h after *V. splendidus* stimulation, which was 3.56-fold (*p* < 0.05), 4.96-fold (*p* < 0.05), 2.41-fold (*p* < 0.05), 1.61-fold (*p* < 0.05), and 2.84-fold (*p* < 0.05) that in the control group, respectively (Figure S2C). Similarly, the expression level of *Cg*VEGFR also increased significantly at 6, 12, and 24 h after *V. splendidus* stimulation, which was 3.81-fold (*p* < 0.05), 2.69-fold (*p* < 0.05), and 6.39-fold (*p* < 0.01) of that in the control group, respectively (Figure S2C).

### 3.5. CgVEGF Interacts with Its Specific Receptor CgVEGFR

The recombinant proteins of the PDGF domain in *Cg*VEGF and the extracellular domain of *Cg*VEGFR were expressed in *E. coli* Transetta DE3 and analyzed by 12% SDS-PAGE, respectively. Two distinct bands with molecular weights of 40 kDa and 103 kDa were observed, and these were consistent with the predicted molecular weight of the PDGF domain of *Cg*VEGF with a GST tag (Figure S3A) and the extracellular domain of *Cg*VEGFR with a Trx tag (Figure S3B), respectively.

The interaction between r*Cg*VEGF and r*Cg*VEGFR was determined by BLI assay on Octet K2 (ForteBio). The biotinylated r*Cg*VEGF was immobilized onto biosensors, and r*Cg*VEGFR was allowed to interact with the immobilized samples. A global fit of the multi-concentration data yielded a KD value of 879 nM, which showed the binding affinity of the interaction between r*Cg*VEGF and r*Cg*VEFGR (Figure S3C). As a control, there was no binding signal when rTrx reacted with the biotin-labeled r*Cg*VEGF.

### 3.6. VEGF Promotes the Proliferation of Hematopoietic Stem Cells and Circulating Hemocytes

**Figure 4:**
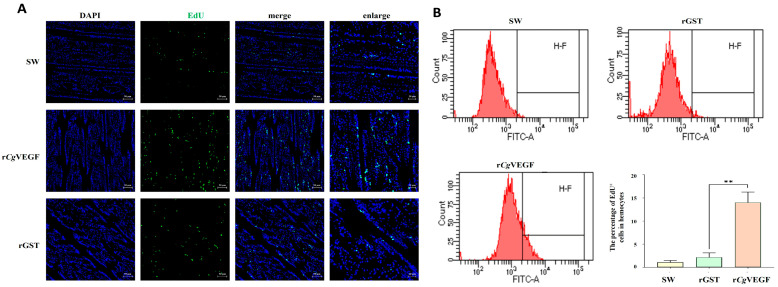
The EdU-positive cells in the G2–G3 sector and hemocytes after r*Cg*VEGF stimulation. (**A**) The newborn cells in the G2–G3 sector after r*Cg*VEGF stimulation were detected by immunofluorescence. The EdU-positive signals are shown in green and the DAPI shows in blue fluorescence signals. Different channels are indicated in different panels. And the right panel shows a magnified image. (**B**) The EdU-positive signals indicating the renewal of circulating hemocytes determined by flow cytometry. The statistical analysis of the percentage of EdU-positive cells in hemocytes. Vertical bars represent the mean ± S.D. (N = 3). The asterisk indicates significant differences (** *p* < 0.01, Duncan).

To investigate the possible role of *Cg*VEGF in hematopoiesis, the newborn cells in the G2–G3 sectors and the circulating hemolymph of oysters after r*Cg*VEGF stimulation were detected by immunofluorescence and flow cytometry assays, respectively. The newborn cells labeled with EdU (EdU^+^) were observed in green, which were mainly distributed in the tubule lumen regions of G2–G3 sector, and colocalized with the blue signals of DAPI (Figure 4A). After r*Cg*VEGF stimulation, the EdU-positive signals increased, and they were stronger than that in the rGST group (Figure 4A).

The newborn cells in the circulating hemolymph were examined by flow cytometry. The EdU-positive signals were observed, and the percentage of EdU^+^ cells in the total circulating hemocytes increased significantly at 6 h after r*Cg*VEGF stimulation (13.0%), which was 6.46-fold of that in the control rGST group (2.01%, *p* < 0.01) (Figure 4B).

### 3.7. VEGF-VEGFR Promotes the Proliferation of Hematopoietic Stem Cells and Circulating Hemocytes Through the MAPK Pathway

**Figure 5:**
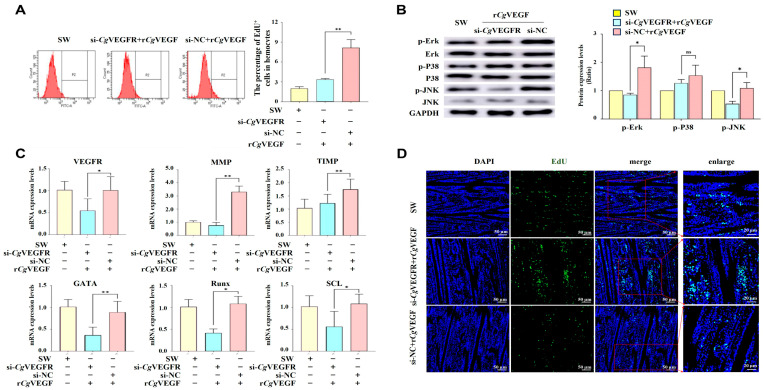
The changes in EdU^+^ cells’ percentages in total hemocytes and the G2–G3 sector, and the phosphorylation level of MAPK pathway proteins in the G2–G3 sector in si-*Cg*VEGFR oysters following r*Cg*VEGF stimulation. (**A**) The EdU-positive signals indicating the renewal of circulating hemocytes determined by flow cytometry. The statistical analysis of the percentage of EdU^+^ cells in hemocytes. (**B**) The phosphorylation levels of ErK, P38, and JNK in the G2–G3 sector in the si-*Cg*VEGFR oysters after r*Cg*VEGF stimulation detected using Western blotting. The gray values analysis for the phosphorylation levels of MAPK signaling pathway. (**C**) The mRNA transcripts of *Cg*VEGFR, hematopoietic transcription factors (*Cg*GATA, *Cg*Runx and *Cg*SCL), and cell migration-related molecules (*Cg*MMP and *Cg*TIMP) in the G2–G3 sector after si-*Cg*VEGFR treatment examined by RT-qPCR. The oysters that received si-NC injection were employed as the control (si-NC group). (**D**) The EdU^+^ cells in the G2–G3 sector of si-*Cg*VEGFR oysters after r*Cg*VEGF stimulation detected by tissue immunofluorescence. The positive signals of EdU labeled with Alexa Fluor 488 are observed in green and the nuclei dyed by DAPI are shown in blue. Different channels are shown as indicated in different panels. The right panel shows a magnified image. Vertical bars represent the mean ± S.D. (N = 3). The asterisks indicate significant differences (* *p* < 0.05, ** *p* < 0.01, Duncan). ns indicate no significant difference.

To recognize the possible function of *Cg*VEGFR in r*Cg*VEGF-induced cell proliferation, the newborn hemocytes were examined by flow cytometry after the expression of *Cg*VEGFR was interfered with in vivo with specific siRNA. The expression level of *Cg*VEGFR was significantly down-regulated after the injection with si-*Cg*VEGFR, which was 0.35-fold of that in the control group (*p* < 0.05). After r*Cg*VEGF stimulation, the expression level of *Cg*VEGFR was significantly down-regulated in si-*Cg*VEGFR + r*Cg*VEGF group, which was 0.27-fold of that in the control si-NC + r*Cg*VEGF group (*p* < 0.01). The percentage of EdU^+^ cells in circulating hemocytes increased significantly in the si-*Cg*VEGFR + r*Cg*VEGF group, which was 0.53-fold of that in the control si-NC + r*Cg*VEGF group (*p* < 0.01) (Figure 5A).

Meanwhile, the phosphorylation level of ErK, P38 and JNK; the mRNA expression level of *Cg*GATA, *Cg*Runx, *Cg*SCL, *Cg*MMP, and *Cg*TIMP; and the EdU-positive signals in G2–G3 sector of the gills were examined in the si-*Cg*VEGFR oysters after r*Cg*VEGF stimulation to further explore the possible role of *Cg*VEGF-VEGFR-MAPK pathways in regulating cell proliferation and migration in the hematopoietic site. The protein abundance of *Cg*VEGFR, p-*Cg*ErK, and p-*Cg*JNK in the G2–G3 sector of the si-*Cg*VEGFR oysters decreased significantly at 6 h after r*Cg*VEGF stimulation, which was 0.54-fold (*p* < 0.05), 0.47-fold (*p* < 0.05) and 0.50-fold (*p* < 0.05) of that in the si-NC group, respectively (Figure 5B). While no significant change in p-*Cg*P38 level was observed after r*Cg*VEGF stimulation. Moreover, the mRNA expression of hematopoietic transcription factors (*Cg*GATA, *Cg*Runx and *Cg*SCL) decreased at 6 h after r*Cg*VEGF stimulation, and were 0.41-fold (*p* < 0.01), 0.39-fold (*p* < 0.05), and 0.52-fold (*p* < 0.05) compared with that in si-NC + r*Cg*VEGF, respectively (Figure 5C). The expression level of cell migration-associated molecules (*Cg*MMP) in si-*Cg*VEGFR + r*Cg*VEGF decreased significantly (0.23-fold, *p* < 0.01), while the mRNA expression of cell migration-associated molecules (*Cg*TIMP) increased significantly (1.43-fold, *p* < 0.01) after r*Cg*VEGF stimulation. An immunohistochemistry assay was performed to further investigate the newborn cells labeled with EdU in the G2–G3 sector of the gill. The green EdU signals were observed in the tubule lumen regions of gill vessels, and the positive signals were more intensive in the si-*Cg*VEGFR + r*Cg*VEGF group compared to those in the si-NC + r*Cg*VEGF group (Figure 5D).

### 3.8. VEGF-VEGFR-MAPK Pathway Enhances the Proliferation of Hematopoietic Stem Cells and Circulating Hemocytes in a VEGF-Dependent Manner

**Figure 6:**
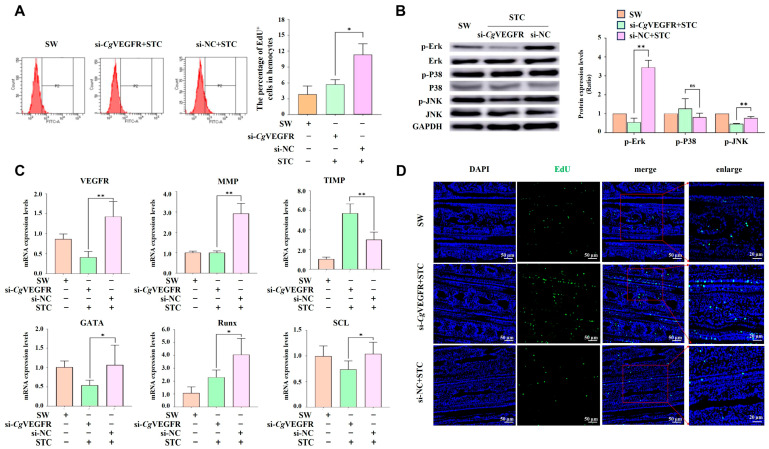
The changes in EdU^+^ cells’ percentages in total hemocytes and the G2–G3 sector, and the phosphorylation level of MAPK pathway proteins in the G2–G3 sector in si-*Cg*VEGFR oysters following STC stimulation. (**A**) The percentage of EdU^+^ hemocytes detected by flow cytometry. The percentage of EdU^+^ hemocytes in oysters after the stimulation with seawater, si-NC + STC, or si-*Cg*VEGFR + STC. (**B**) The phosphorylation level of MAPK pathway proteins ErK, P38, and JNK in the G2–G3 sector in si-*Cg*VEGFR after STC stimulation detected using Western blotting (left). The gray values for the protein abundance analyzed by ImageJ (right). (**C**) The mRNA transcripts of hematopoietic factors (*Cg*GATA, *Cg*Runx and *Cg*SCL) and cell migration-related genes (*Cg*MMP and *Cg*TIMP) in the G2–G3 sector of si-*Cg*VEGFR oysters following STC stimulation examined by RT-qPCR. (**D**) The EdU^+^ cells in the G2–G3 sector of si-*Cg*VEGFR oysters following STC stimulation detected by tissue immunofluorescence. The positive signals of EdU labeled with Alexa Fluor 488 are shown in green and the nuclei dyed by DAPI are shown in blue. Different channels are shown as indicated in different panels. The right panel shows a magnified image. Vertical bars represent the mean ± S.D. (N = 3). The asterisks indicate significant differences (* *p* < 0.05, ** *p* < 0.01, Duncan). ns indicate no significant difference.

The percentage of EdU^+^ cells in circulating hemocytes; the phosphorylation levels of the MAPK pathway; the transcription level of *Cg*GATA, *Cg*Runx, *Cg*SCL, *Cg*MMP, and *Cg*TIMP; and the presence of EdU-positive signals in the G2–G3 sector of the gill were examined in si-*Cg*VEGFR oysters after STC (VEGF activator) stimulation. The percentage of EdU-positive cells in circulating hemocytes increased significantly in the si-*Cg*VEGFR + STC group, which was 0.50-fold of that in the si-NC + STC control group (*p* < 0.05) (Figure 6A). After STC stimulation, the mRNA expression level of *Cg*VEGFR in the G2–G3 sector of the gill was significantly reduced (0.29-fold, *p* < 0.01) in the si-*Cg*VEGFR group compared with that in the si-NC group (Figure 6C). Additionally, the phosphorylation levels of p-*Cg*ErK and p-*Cg*JNK in the si-*Cg*VEGFR + STC group decreased significantly (0.16-fold and 0.62-fold, *p* < 0.01, respectively) compared with those in the si-NC + STC group. There was no significant difference observed in the expression level of p-*Cg*P38 (Figure 6B).

Furthermore, the mRNA expression levels of hematopoietic transcription factors *Cg*GATA, *Cg*Runx and *Cg*SCL decreased significantly (0.51-fold, 0.57-fold, and 0.71-fold, *p* < 0.05, respectively) in the si-*Cg*VEGFR + STC group compared with those in the si-NC + STC group (Figure 6C). Additionally, the transcripts of *Cg*MMP decreased significantly (0.34-fold, *p* < 0.05) in the si-*Cg*VEGFR + STC group, while the transcripts of *Cg*TIMP increased significantly (1.89-fold, *p* < 0.05) compared to those in the si-NC + STC group (Figure 6C).

The immunohistochemistry assay showed that EdU labeling signals were stronger in the tubule lumen regions of gill vessels in the si-*Cg*VEGFR + STC group compared to those in the si-NC + STC group (Figure 6D).

### 3.9. The VEGF-VEGFR-MAPK Pathway Enhances the Proliferation and Migration of Hematopoietic Stem Cells and Circulating Hemocytes

**Figure 7:**
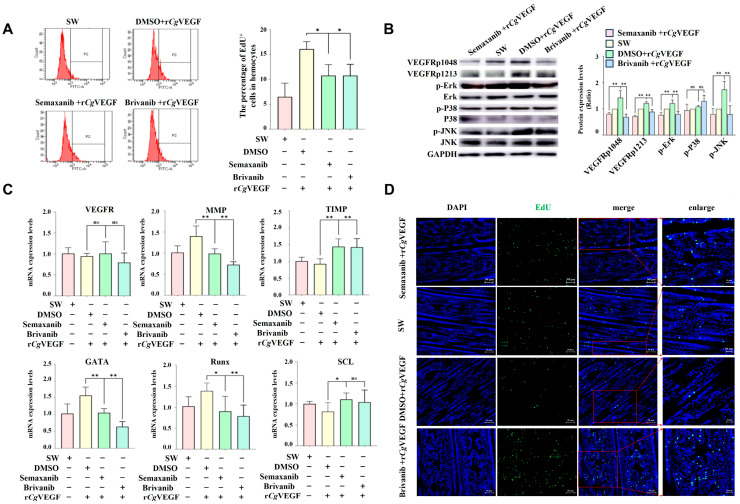
The expression of *Cg*VEGFR, MAPK pathway genes, cell proliferation- and cell migration-related genes, and EdU^+^ cells in the G2–G3 sector and in the hemocytes of VEGFR-inhibited oysters after r*Cg*VEGF stimulation. (**A**) The rate of EdU^+^ hemocytes in oysters after stimulation with seawater, Semaxanib, Brivanib, or DMSO detected by flow cytometry. (**B**) The phosphorylation level of MAPK pathway proteins ErK, P38, and JNK in the G2–G3 sector of *Cg*VEGFR-inhibited oysters after r*Cg*VEGF stimulation determined by Western blotting. The gray values for the protein abundance analyzed by ImageJ. (**C**) The mRNA transcripts of hematopoietic factors (*Cg*GATA, *Cg*Runx, and *Cg*SCL) and cell migration-related genes (*Cg*MMP and *Cg*TIMP) in the G2–G3 sector of *Cg*VEGFR-inhibited oysters after r*Cg*VEGF stimulation examined by RT-qPCR. The oysters that were injected with DMSO were employed as the control (DMSO group). (**D**) The EdU^+^ cells at the G2–G3 sector in si-*Cg*VEGFR oysters after r*Cg*VEGF stimulation detected by tissue immunofluorescence. The positive signals of EdU labeled with Alexa Fluor 488 are shown in green and the nuclei dyed by DAPI are shown in blue. Different channels are shown in different panels. The right panel shows a magnified image. Vertical bars represent the mean ± S.D. (N = 3). The asterisk indicates significant differences (* *p* < 0.05, ** *p* < 0.01, Duncan). ns indicate no significant difference.

The proliferation and migration capability of cells in the hematopoietic site were examined in VEGFR-inhibited oysters after the treatment with r*Cg*VEGF and the VEGFR inhibitors Semaxanib and Brivanib. The percentage of EdU-positive cells in circulating hemocytes in the Semaxanib + r*Cg*VEGF group and the Brivanib + r*Cg*VEGF group were 0.67-fold (*p* < 0.05) and 0.67-fold (*p* < 0.05) of that in the DMSO + r*Cg*VEGF group (Figure 7A). The phosphorylation level o*f Cg*VEGFR protein at Tyr1048 and Tyr1213 sites in the Semaxanib + r*Cg*VEGF group and the Brivanib + r*Cg*VEGF group decreased significantly, which was 0.47-fold (*p* < 0.05 and 0.50-fold (*p* < 0.05), 0.48-fold (*p* < 0.05) and 0.73-fold (*p* < 0.05) of that in the DMSO + r*Cg*VEGF group, respectively (Figure 7C). The levels of p-*Cg*ErK and p-*Cg*JNK in Semaxanib + r*Cg*VEGF group and Brivanib + r*Cg*VEGF group decreased significantly, to 0.01-fold (*p* < 0.01) and 0.01-fold (*p* < 0.01), 0.01-fold (*p* < 0.01) and 0.02-fold (*p* < 0.01) of those in the DMSO + r*Cg*VEGF group, respectively. No significant change in the p-*Cg*P38 level was observed in the Semaxanib + r*Cg*VEGF group or the Brivanib + r*Cg*VEGF group. The mRNA expression level of *Cg*VEGFR in the G2–G3 sector in the Semaxanib + r*Cg*VEGF group and the Brivanib + r*Cg*VEGF group was 1.00-fold (*p* > 0.05) and 0.99-fold (*p* > 0.05) of that in the DMSO + r*Cg*VEGF group (Figure 7B). Moreover, the mRNA expression of hematopoietic transcription factors (*Cg*GATA and *Cg*Runx) decreased after Semaxanib and Brivanib treatments, and was found to be 0.67-fold (*p* < 0.01) and 0.65-fold (*p* < 0.05), 0.41-fold (*p* < 0.01) and 0.67-fold (*p* < 0.01) compared with that in DMSO + r*Cg*VEGF, respectively. The mRNA expression of *Cg*SCL was 1.35-fold (*p* < 0.05) and 1.20-fold (*p* > 0.05 of that in the DMSO + r*Cg*VEGF group. The expression level of cell migration-associated molecules (*Cg*MMP) in the Semaxanib + r*Cg*VEGF group (0.70-fold, *p* < 0.01) and Brivanib + r*Cg*VEGF group (0.52-fold, *p* < 0.01) decreased significantly, while the mRNA expression of cell migration-associated molecules (*Cg*TIMP) increased significantly (1.56- and 1.54-fold, *p* < 0.01) in the DMSO + r*Cg*VEGF group (Figure 7C).

An immunohistochemistry assay was performed to further investigate the newborn cells labeled with EdU in the G2–G3 sector of the gill. The green signals of EdU were observed in the tubule lumen regions of gill vessels, and the positive signals were more intensive in the Semaxanib + r*Cg*VEGF group and Brivanib + r*Cg*VEGF group compared to those in the DMSO + r*Cg*VEGF group (Figure 7D).

## 4. Discussion

Hematopoiesis is a continuous process of immune cell production, which is orchestrated by thousands of genes that respond to extracellular signals by guiding cell fate decisions. During hematopoiesis, newborn immune cells are generated from the HSCs residing in the hematopoietic sites or hematopoietic tissues [23]. In parallel with the evolution from aquatic to terrestrial life, blood cell production migrates from earlier sites in the thorax and abdomen to the bone marrow cavity [24]. For the primitive marine bivalves, the potential hematopoietic sites and tissues have not been clearly defined, although it has been suspected that hematopoiesis occurs in gills. In the present study, the proximal hinge region of the oyster gill was characterized as a potential hematopoietic site that may be responsible for the production and subsequent release of hemocytes into the circulation, and demonstrated that the VEGF-VEGFR-MAPK pathway orchestrated the production and subsequent migration of hemocytes into the circulation, suggesting a functional parallel to the thymic precursor in jawless vertebrates.

Hematopoietic tissue is typically characterized by the presence of HSCs. All the terminally differentiated immune cells originated from HSCs through a progressively restricted process of cell fate determination [25]. The fate of HSCs is orchestrated by a myriad of extrinsic signals, including cytokines and reactive oxygen species. These signals are subsequently interpreted and integrated by intrinsic factors, such as hematopoietic transcription factors, to establish specific gene regulatory programs that dictate cell fate. The hematopoietic transcription factors and hematopoietic growth factors are usually more highly expressed in the hematopoietically active regions [5,26]. GATA-binding protein 3 (GATA3), stem cell leukemia (SCL), and runt-related protein (Runx), PCNA, Astakine, and IL-17 have been previously identified as important hematopoiesis-related factors in oysters [27]. In the present study, hematopoiesis transcription factors (*Cg*GATA, *Cg*SCL and *Cg*Runx), cycle-related proteins (*Cg*PCNA and *Cg*CDK2), and hematopoietic growth factor (*Cg*Astakine) were found to be relatively highly expressed at the proximal region of the hinge (G2–G3 sector) of the gill compared to the other sectors. The relatively high expression levels of these genes in the G2–G3 sector suggested that this site might be essential for hemocyte proliferation in oysters. Interestingly, most of the immune effectors (such as defensin superfamily members and inflammatory cytokines) exhibited lower expression levels at the distal region of the hinge in oyster gill. A prevailing model posits that HSC fate determination is driven by a dynamic transcriptional switch. The up-regulation of lineage-specific transcription factors promotes differentiation along a particular pathway, while simultaneously repressing alternative lineages through the down-regulation of their requisite transcriptional regulators [28]. HSCs in mammals are morphologically characterized by small, round to ovoid cytomorphology with a high nuclear/cytoplasmic ratio [29]. Similarly to mammalian HSCs, a group of small round cells with a high nuclear/cytoplasmic ratio was identified in the G2–G3 sector of oyster gills. Consistently, the cells in hematopoietic tissue in crab *Eriocheir sinensis* were also reported to be less granular with a big nucleus [30]. The high expression of hematopoietic and stemness-related factors, and the presence of morphologically HSC-like cells in the proximal region of the hinge in gills (G2–G3 sector), suggested that this site might be endowed with hemocyte repopulating activity similar to the hematopoietic tissue in mammals.

The functional integrity and long-term maintenance of hematopoietic tissue hinge on the precise equilibrium of HSC self-renewal, quiescence, and differentiation. Hematopoietic tissue harbors the highest self-renewal potential of all immune cells [31]. The preservation of long-term hematopoietic function relies on maintaining most HSCs in a quiescent state, which is a protective strategy designed to defend against stem cell exhaustion. These dormant, slow-cycling cells are classically identified as label-retaining cells (LRCs) [32]. Conversely, HSCs possess a rapid adaptability to stressors like infection and inflammation, mounting a response that involves exiting quiescence, increasing cell cycling, and generating hematopoietic progenitors. In a model of salmonella infection in zebrafish, increased neutrophil production was found during infection, which was the consequence of HSCs’ expansion in hemopoietic tissue [33]. Crustacean hemocytes were reported to be released continuously from specialized hematopoietic tissue during infection [34]. In mammals, HSCs reversibly cycle between a dormant state and active self-renewal, exiting quiescence not stochastically but in a regulated manner to tackle hematopoietic stress, and subsequently returning to dormancy upon the restoration of homeostasis [35]. In the present study, a population of small round-shaped cells was identified in the G2–G3 sector of gill, and the EdU-positive newborn cells were observed at the tubule lumen region of gill vessels. The EdU signals in G2–G3 sector became stronger and were maintained at a higher level even at 240 h after *V. splendidus* stimulation. They were partially colocalized with stemness transcription factor *Cg*SOX2. These results suggested that gill filaments in the G2–G3 sector possess the capacity for self-renewing hemocytes, supporting the hypothesis that this area serves as a hematopoietic site in oysters.

The hematopoietic microenvironment within hematopoietic tissue forms a highly organized three-dimensional structure to support hematopoiesis, which is mainly constituted by a cellular network and its products (including the extracellular matrix, cytokines, chemokines, and growth factors) [36]. Growing evidence has demonstrated that VEGF, a principal regulator of angiogenesis, is also crucial for the survival and repopulation of HSCs. Human VEGF is highly expressed in richly vascularized tissue, including the brain choroid plexus, lung alveoli, kidney glomeruli, and heart, while its receptors (VEGFR1 and VEGFR2) are usually expressed in immature hematopoietic cells with repopulating activity to play vital roles in cardiovascular, hematopoietic, and lymphatic development [37]. In the present study, *Cg*VEGF and *Cg*VEGFR mRNA were found to be significantly higher expressed in the G2–G3 sector. Their expression levels in the G2–G3 sector of the gill increased significantly after *V. splendidus* stimulation, suggesting that they were involved in hematopoiesis against immune stimulation. Similar results have also been reported in humans [38], shrimp *Litopenaeus vannamei* [39], carb *E. sinensis* [40], Drosophila [41], cephalopoda *Idiosepius paradox* [42], and Cnidarians [43]. These results suggest that *Cg*VEGF and *Cg*VEGFR play a vital role in hemocyte proliferation in oysters.

The interaction between VEGF and its target cell surface receptor transmits signals to drive cellular responses, including cell proliferation, survival, migration, and deposition [44]. For instance, VEGF was reported to induce the proliferation of coelomocytes or hemocytes in sea cucumber *Apostichopus japonicus* [36] and Drosophila [11]. In our study, the EdU-positive cells in both G2–G3 sectors of the gill increased after the oysters received an injection with r*Cg*VEGF, while decreased dramatically when the expression of *Cg*VEGFR was inhibited, indicating that *Cg*VEGF was able to positively regulate the production of newborn hemocytes in oysters. In mammals, VEGFR is also required for the recruitment of hematopoietic precursors and the migration of monocytes and macrophages [28]. In invertebrates, VEGF/VEGFR signaling is responsible for guiding cell migration in sea urchin [45], Drosophila [46], and crayfish [12]. In the oyster, the percentage of EdU-positive cells in the circulating hemolymph fluctuated after the injection of r*Cg*VEGF or VEGF inducer, as well as the inhibition of *Cg*VEGFR with the siRNA or VEGFR inhibitors, suggesting the responsibility of VEGF/VEGFR in the migration of newborn hemocytes to circulating hemolymph in oysters.

As a member of the tyrosine kinase family, VEGFR is involved in various cellular processes by activating the downstream signaling pathway. It has been reported that the binding of VEGF to its receptor activates the MAPK pathway, resulting in the proliferation, migration, and invasion of endothelial cells [47]. In Drosophila, the Ras/MAPK signaling elicited by PVR and its ligand is involved in cell proliferation and migration during embryogenesis [48]. In the present study, the phosphorylation levels of Erk and JNK, the mRNA expression levels of hematopoietic (*Cg*PCNA, *Cg*CDK2, *Cg*GATA, and *Cg*Runx) and cell migration-related (*Cg*MMT) genes as well as the percentage of EdU-positive cells all increased and aggregated in the G2–G3 sector after the injection with r*Cg*VEGF or VEGF inducer or when the expression of *Cg*VEGFR was inhibited by siRNA or VEGFR inhibitors. These results demonstrated that *Cg*VEGF-VEGFR signaling promoted the proliferation and migration of newborn hemocytes by activating the MAPK pathway during oyster hematopoiesis, thereby revealing a conserved regulatory mechanism in hematopoietic control.

## 5. Conclusions

In summary, the proximal region of the hinge in the gill was refined as the potential hematopoietic site of the oyster with higher expression levels of the hematopoietic-related genes, the residence of a large number of newborn cells, and a few stem-like cells. *Cg*VEGF and *Cg*VEGFR were highly expressed in the potential hematopoietic site, and were significantly up-regulated post *V. splendidus* stimulation. *Cg*VEGFR was able to interact with *Cg*VEGF to mediate the production and migration of newborn hemocytes to the circulating hemolymph by activating the MAPK pathway in oysters, which provided novel insights into the regulation mechanism of hematopoiesis in primitive invertebrates.

## Data Availability

All study data are included in the article. For other information and Supplementary Materials related to this project, please contact the corresponding authors.

## Supplementary Materials

The following supporting information can be downloaded at https://www.mdpi.com/article/10.3390/cells14181446/s1, Figure S1: The structural characteristics of G2 to G3 sector. A. The schematic representation of the G2–G3 sector in gills and the regions examined by hematoxylin-eosin staining. B. The EdU staining of the G2–G3 sector in gill; Figure S2: The mRNA expression profiles of *Cg*VEGF and *Cg*VEGFR. A. The expression profiles of *Cg*VEGF and *Cg*VEGFR mRNA in different tissues examined by RT-qPCR analysis. Amu: adductor muscle; Man: mantle; Lpa: labial palp; Dgl: digestive gland; Hae: haemocytes; Gil: gill; Go: gonad. B. The mRNA transcripts of *Cg*VEGF and *Cg*VEGFR in different sectors of gill. C. The expression levels of *Cg*VEGF and *Cg*VEGFR in the G2–G3 sector at 0, 3, 6, 12, 24, 48, and 72 h after *V. splendidus* stimulation, with the seawater stimulation group as control. Vertical bars represent the mean ± S.D. (N = 3). The asterisk (*) indicates significant differences (* *p* < 0.05, Duncan); Figure S3: Interaction between r*Cg*VEGF and r*Cg*VEGFR in vitro. A. SDS-PAGE of r*Cg*VEGF. B. SDS-PAGE of r*Cg*VEGFR. Lane M: Protein molecular standard; Lane 1: Negative control of plasmid vector (without induction); Line 2: The induced recombinant bacteria lysate of plasmid vector; Lane 3: Negative control of r*Cg*VEGF or r*Cg*VEGFR (without induction); Lane 4: The induced recombinant bacteria lysate of r*Cg*VEGF or r*Cg*VEGFR; Lane 5: Purified r*Cg*VEGF or r*Cg*VEGFR. C. The combination of different concentrations of r*Cg*VEGFR subunit (colour lines) with r*Cg*VEGF. The black lines are from model fits; Table S1: Sequences of the primers used in this study.

## Abbreviations

HSCs	Hematopoietic stem cells.
G	Gill.
SW	Seawater.
VS	*Vibrio splendidus.*
