# Supplementary material for: VEGF-VEGFR Signaling Mechanism Directs the Migration of Newborn Hemocytes from the Hematopoietic Site of Oyster Crassostrea gigas
Source: Cells. 2025 Sep 16;14(18):1446. doi: 10.3390/cells14181446 (PMC12468279; doi:10.3390/cells14181446)
Supplement: Supplementary file 1 [file cells-14-01446-s001.zip › cells-3817504-Table S1.pdf]

Table of Contents

The proximal region of hinge in the gill has been refined as the potential haematopoietic site of oyster with the higher expression levels of the haematopoietic-related molecule and the residence of large number of newborn cells and a few stem-like cells. And *Cg*VEGF-VEGFR-MAPK signaling pathway induced the migration of newborn haemocytes from this site to the circulating haemolymph.

Supplementary Table

Table S1. Sequences of the primers used in this study.

Primer	Sequence (5'-3')
si-*Cg*VEGFR	gacaggttatcaacgaaacttct
*Cg*EF-RT-F	AGTCACCAAGGCTGCACAGAAAG
*Cg*EF-RT-R	TCCGACGTATTTCTTTGCGATGT
*Cg*VEGF-RT-F	TGTGAGCTGCGACAAGGCGT
*Cg*VEGF-RT-R	TCGACAGGGGGCGTTAGGGA
*Cg*VEGFR-RT-F	CTGCCCTTGGGTGACCTGGA
*Cg*VEGFR-RT-R	CAGATCTCTGACCAGGAGCTATCAT
*Cg*Runx-RT-F	TGGCGTTCCAACAAGACATTA
*Cg*Runx-RT-R	AAACTTTTTCCACGACCGCTA
*Cg*GATA-3-RT-F	CTGTGGAGGAGGGATGGGACC
*Cg*GATA-3-RT-R	TCTTTCTTCATTGTAAGCGGGCG
*Cg*SCL-RT-F	CTGAACATGGTGGTGTTTACTGAAG
*Cg*SCL-RT-R	TAAAATAGACACGACCATCCATCC
*Cg*SOX2-RT-F	CCAGCAAAAAAGACGCAGACAGAGT
*Cg*SOX2-RT-R	TCGGCTTCGGATAATAATTTCCACT
*Cg*defh1-RT-F	ATTAGCCGTTCTTCTGATGG
*Cg*defh1-RT-R	GCTCTACAACCGATGGACCT
*Cg*defh2-RT-F	TGGTCGTTCTCCTGATGGTTT
*Cg*defh2-RT-R	CTGCGTCACAGTAGCCCG
*Cg*BigDef1-RT-F	GGTTTCGCCTGCTTCCAT
*Cg*BigDef1-RT-R	GCACCACCCTCGGTTGTTAG
*Cg*MMP-F	TACGTGACAAGGACACTGCC
*Cg*MMP-R	CGAGACGCTGCTCCACTTAT
*Cg*TIMP-F	GTACTCCGTGGAACTGTGGG
*Cg*TIMP-R	GGAAGTCGCAGTTCTCACCA
*Cg*EF-F	AGTCACCAAGGCTGCACAGAAAG
*Cg*EF-R	TCCGACGTATTTCTTTGCGATGT
