# Supplementary material for: VEGF-VEGFR Signaling Mechanism Directs the Migration of Newborn Hemocytes from the Hematopoietic Site of Oyster Crassostrea gigas
Source: Cells. 2025 Sep 16;14(18):1446. doi: 10.3390/cells14181446 (PMC12468279; doi:10.3390/cells14181446)
Supplement: Supplementary file 1 [file cells-14-01446-s001.zip › Figure legends of Supplementary Files.pdf]

Supplementary Figures

Figure S1. The structural characteristics of G2 to G3 sector. A. The schematic representation of the G2-G3 sector in gills and the regions examined by hematoxylin-eosin staining. B. The EdU staining of the G2-G3 sector in gill.

Figure S2. The mRNA expression profiles of *Cg*VEGF and *Cg*VEGFR. A. The expression profiles of *Cg*VEGF and *Cg*VEGFR mRNA in different tissues examined by RT-qPCR analysis. Amu: adductor muscle; Man: mantle; Lpa: labial palp; Dgl: digestive gland; Hae: haemocytes; Gil: gill; Go: gonad. B. The mRNA transcripts of *Cg*VEGF and *Cg*VEGFR in different sectors of gill. C. The expression levels of *Cg*VEGF and *Cg*VEGFR in the G2-G3 sector at 0, 3, 6, 12, 24, 48, and 72 h after *V. splendidus* stimulation, with the seawater stimulation group as control. Vertical bars represent the mean ± S.D. (N=3). The asterisk (*) indicates significant differences (*$p < 0.05$, Duncan).

Figure S3. Interaction between r*Cg*VEGF and r*Cg*VEGFR *in vitro*. A. SDS-PAGE of r*Cg*VEGF. B. SDS-PAGE of r*Cg*VEGFR. Lane M: Protein molecular standard; Lane 1: Negative control of plasmid vector (without induction); Line 2: The induced recombinant bacteria lysate of plasmid vector; Lane 3: Negative control of r*Cg*VEGF or r*Cg*VEGFR (without induction); Lane 4: The induced recombinant bacteria lysate of r*Cg*VEGF or r*Cg*VEGFR; Lane 5: Purified r*Cg*VEGF or r*Cg*VEGFR. C. The combination of different concentrations of r*Cg*VEGFR subunit (colour lines) with r*Cg*VEGF. The black lines are from model fits.
