# Supplementary figures and images for: VEGF-VEGFR Signaling Mechanism Directs the Migration of Newborn Hemocytes from the Hematopoietic Site of Oyster Crassostrea gigas
Source: Cells. 2025 Sep 16;14(18):1446. doi: 10.3390/cells14181446 (PMC12468279; doi:10.3390/cells14181446)

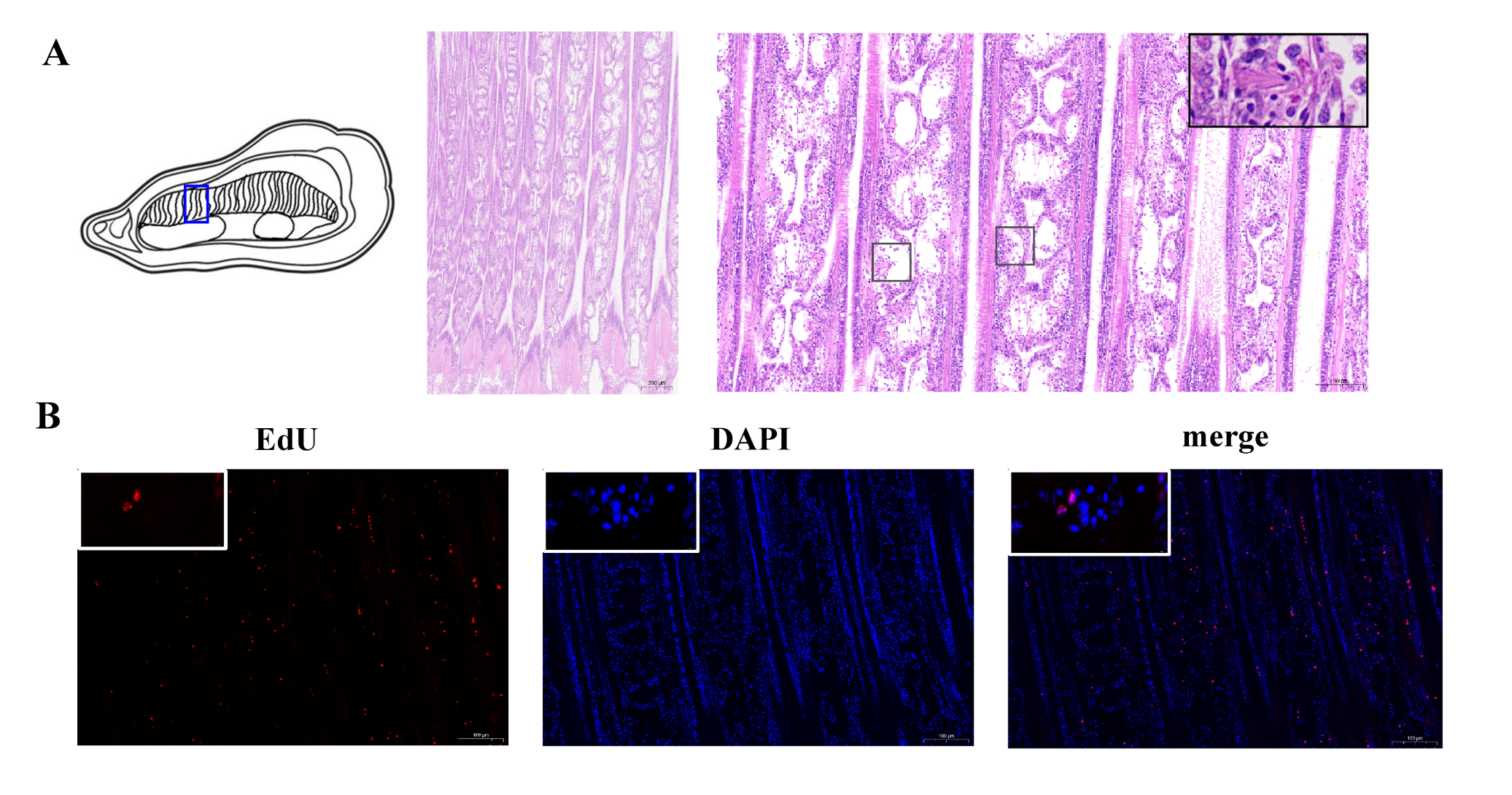

Supplement: Supplementary file 1 [file cells-14-01446-s001.zip › Figure S1.tif]

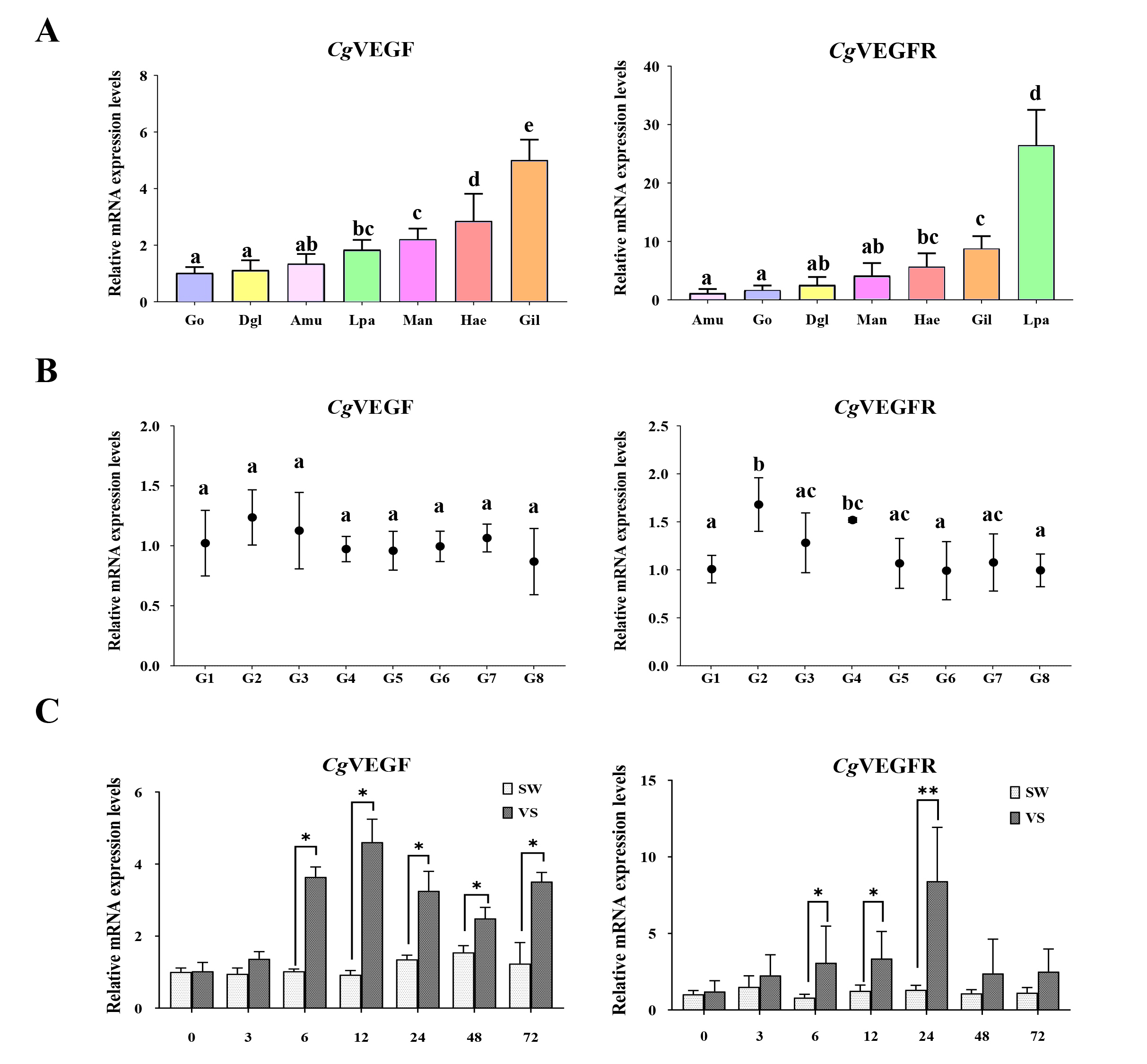

Supplement: Supplementary file 1 [file cells-14-01446-s001.zip › Figure S2.tif]

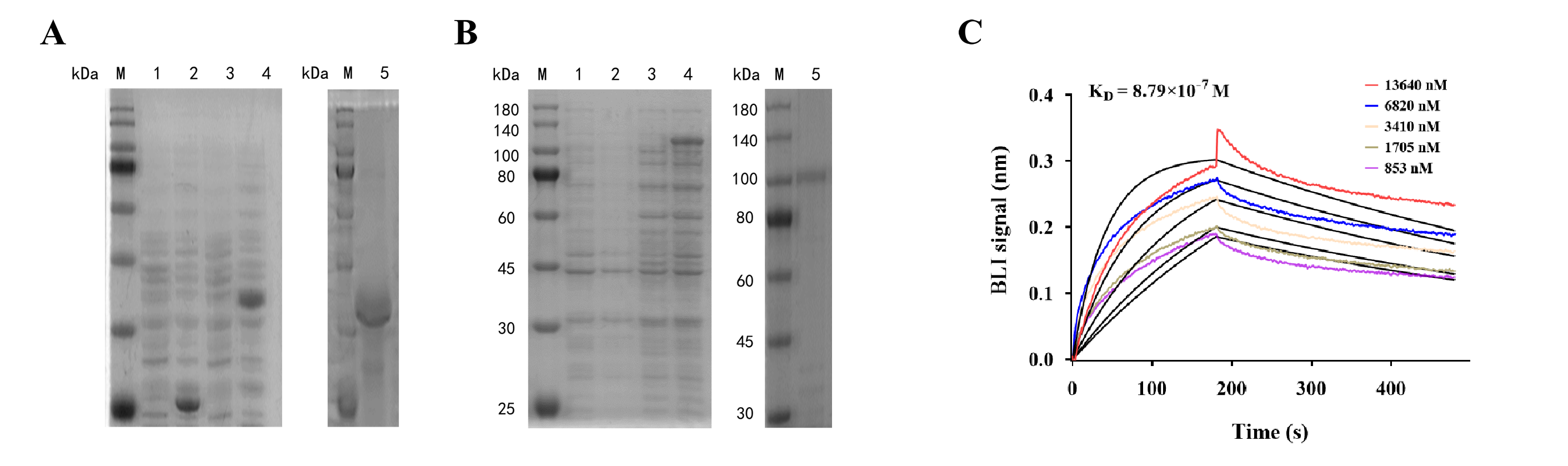

Supplement: Supplementary file 1 [file cells-14-01446-s001.zip › Figure S3.tif]
